# Supplementary material for: amer1 Regulates Zebrafish Craniofacial Development by Interacting with the Wnt/β-Catenin Pathway
Source: Int J Mol Sci. 2024 Jan 5;25(2):734. doi: 10.3390/ijms25020734 (PMC10815499; doi:10.3390/ijms25020734)
Supplement: Supplementary file 1 [file ijms-25-00734-s001.zip › Supplementary Table S1.pdf]

Supplementary Table S1. Sequences of probes used in *in situ* hybridization

Gene	Primer type	Sequence (5'-3')
crestin	Forward	CAGAAGCCCTCATCAGAGAGTTG
	Reverse	GTTGCTTGTCAGGCAGAATCAGG
foxd3	Forward	CCTACTCGTACATCGCCCTC
	Reverse	CCGGGTTAAGGACAGGGAC
dlx2a	Forward	CACAGTTCTGCTTTGCGTCG
	Reverse	CCCAAGTCGGCAGAGTCAAA
barx1	Forward	CTGGGCGGATCAGACTTCTC
	Reverse	GCTTCTCGTGTCCTCTCCTG
sox9	Forward	CCTCGACCCCTACCTGAAGA
	Reverse	GGCGGGAGGTATTGGTCAAA
col2a1a	Forward	TCTGAAGTCCATCAACGGGC
	Reverse	TTTTCCGTCACGCTAAACGC
fgf3	Forward	GAATCTCTGGCTCCGAGGCT
	Reverse	GCCGCTGACTCTCTCTAAGC
tbx1	Forward	GCAGCTGTCCCATTTTTGCG
	Reverse	ACGGCGGTAAATCTGGTCTC
nkx2.3	Forward	TCGTGTTTTCTCGGAGGTGG
	Reverse	GCGCATTAGTGGACGTGTTC
