# Supplementary material for: amer1 Regulates Zebrafish Craniofacial Development by Interacting with the Wnt/β-Catenin Pathway
Source: Int J Mol Sci. 2024 Jan 5;25(2):734. doi: 10.3390/ijms25020734 (PMC10815499; doi:10.3390/ijms25020734)
Supplement: Supplementary file 1 [file ijms-25-00734-s001.zip › Supplementary Table S2.pdf]

Supplementary Table S2. Sequences used in Cas9/gRNA design and validation PCR

Function	Type	Sequence (5'-3')
Cas9/gRNA design*	T7-promoter	TAATACGACTCACTATA
	gRNA target R1	AACCCCGGATGAGATAGACG
	gRNA target R2	GGCTCATTACGACCCTCCAG
	gRNA target R3	GGAGATCGCCACAAGATGTG
	gRNA target R4	AATATTGTTCATAACTTGGG
	Scaffold sequence	GTTTTAGAACTAGAAATAGC
	Tracr rev sequence	AAAAAAAGCACCGACTCGGTGCCAC
Validation PCR	R1-Forward	CATCCTGCTCCTCTGAGAACTT
	R1-Reverse	CATAAAGGGCATCACCACTGTA
	R2-Forward	ATCTCAGGAGTCCATCCAAAAA
	R2-Reverse	ACATCGAATGTTACAATGGCAG
	R3-Forward	CCTCAATGGATAGTTGCTCAAA
	R3-Reverse	TGATGCCACTTCACTGCTACTT
	R4-Forward	ACAATTGCCGTAGTCGTAACCT
	R4-Reverse	CTGGCTACATAAATGCAAAGCA

* The structure of the forward primer is T7-promoter – R1/R2/R3/R4 – Scaffold sequence.
