# Supplementary figures and images for: amer1 Regulates Zebrafish Craniofacial Development by Interacting with the Wnt/β-Catenin Pathway
Source: Int J Mol Sci. 2024 Jan 5;25(2):734. doi: 10.3390/ijms25020734 (PMC10815499; doi:10.3390/ijms25020734)

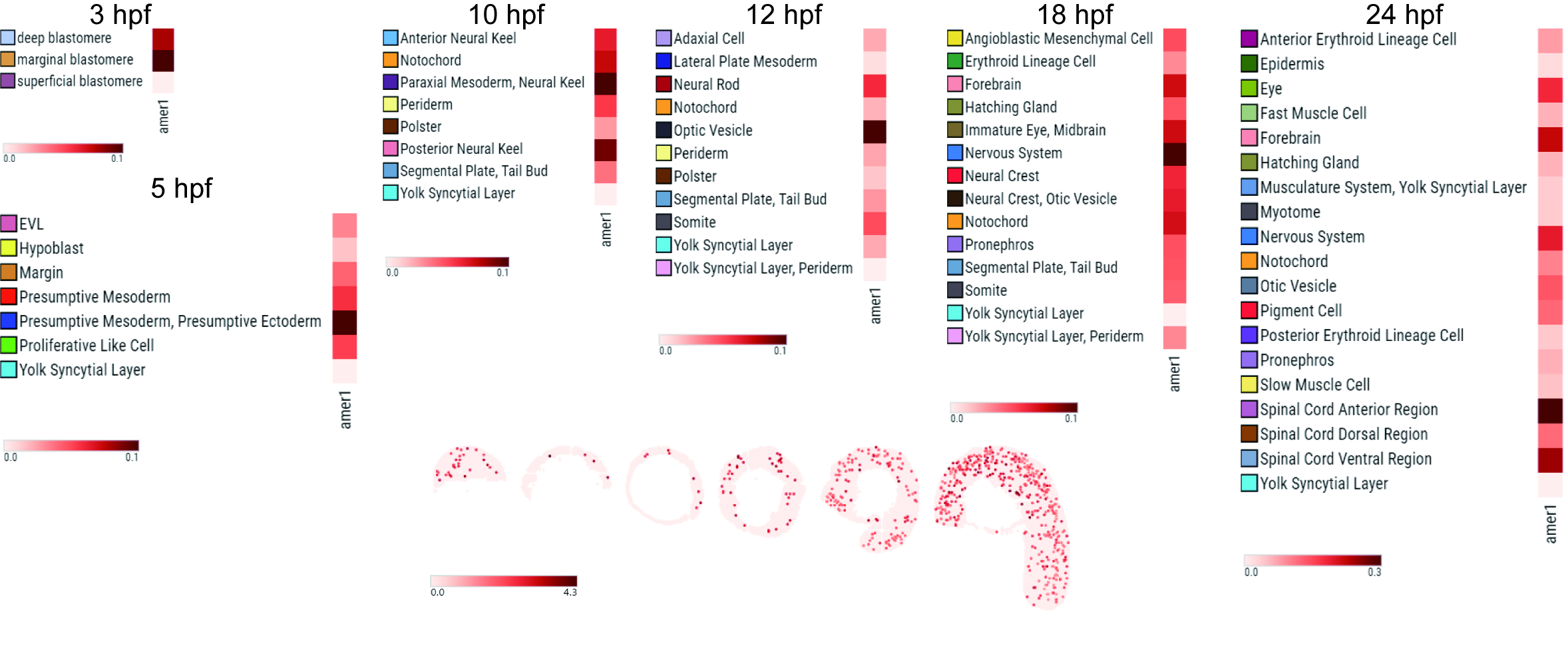

Supplement: Supplementary file 1 [file ijms-25-00734-s001.zip › Supplementary Figure S1.tif]

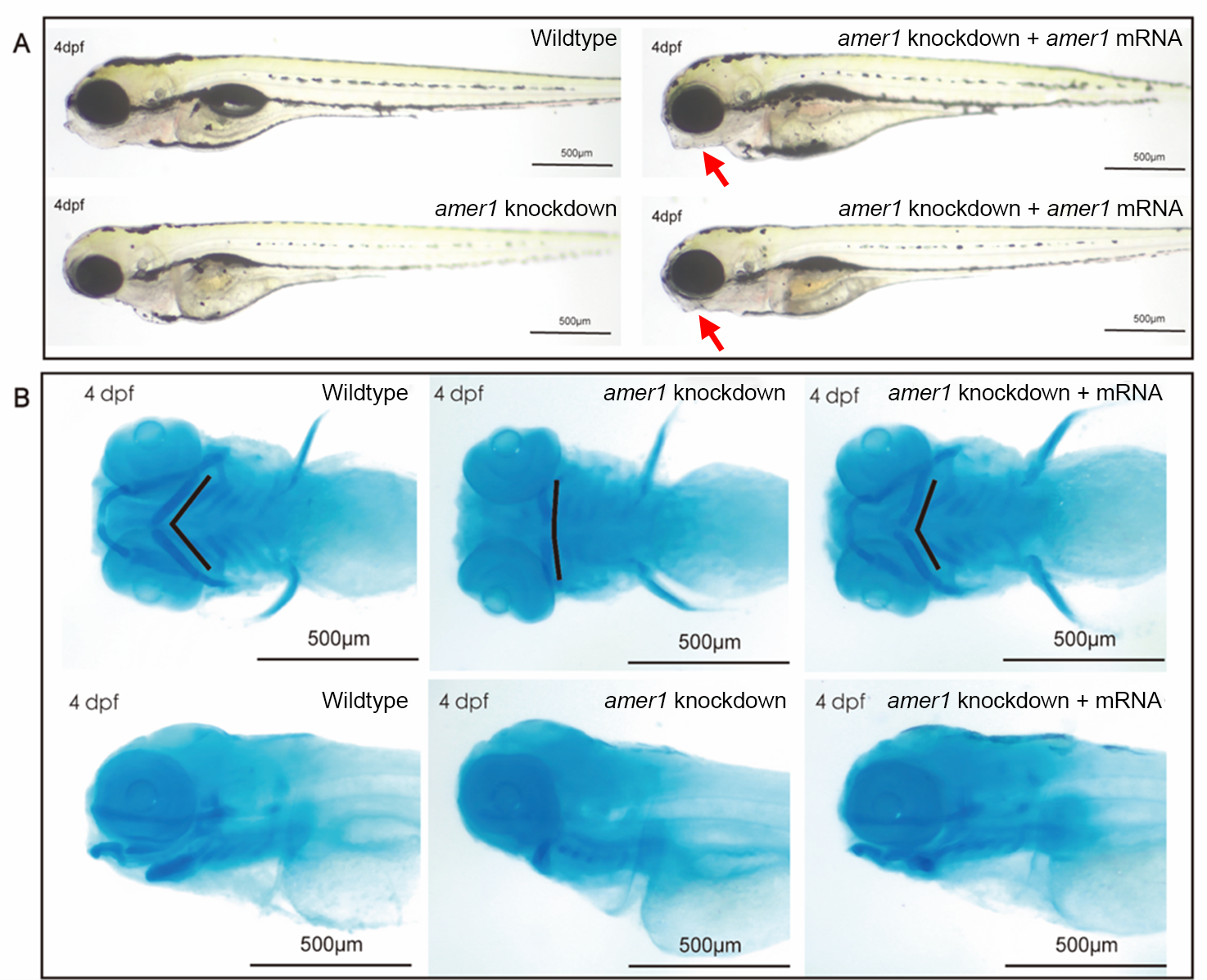

Supplement: Supplementary file 1 [file ijms-25-00734-s001.zip › Supplementary Figure S2.tif]
